# NEXAFS imaging to characterize the physio-chemical composition of cuticle from African Flower Scarab *Eudicella gralli*

**DOI:** 10.1038/s41467-019-12616-5

**Published:** 2019-10-18

**Authors:** Joe E. Baio, Cherno Jaye, Erin Sullivan, Mette H. Rasmussen, Daniel A. Fischer, Stanislav Gorb, Tobias Weidner

**Affiliations:** 1 School of Chemical, Biological and Environmental Engineering, Oregon State University, Corvallis, OR USA; 2 National Institute of Standards and Technology, Gaithersburg, MD USA; 3 Woodland Park Zoo, Seattle, WA USA; 4 Department of Chemistry, Aarhus University, 8000 Aarhus C, Denmark; 5 Functional Morphology and Biomechanics, Zoological Institute, Kiel University, 24118 Kiel, Germany

**Keywords:** Nanoscale biophysics, Biomechanics, Bioinspired materials

## Abstract

The outermost surface of insect cuticle is a high-performance interface that provides wear protection, hydration, camouflage and sensing. The complex and inhomogeneous structure of insect cuticle imposes stringent requirements on approaches to elucidate its molecular structure and surface chemistry. Therefore, a molecular understanding and possible mimicry of the surface of insect cuticle has been a challenge. Conventional optical and electron microscopies as well as biochemical techniques provide information about morphology and chemistry but lack surface specificity. We here show that a near edge X-ray absorption fine structure microscope at the National Synchrotron Light Source can probe the surface chemistry of the curved and inhomogeneous cuticle of the African flower scarab. The analysis shows the distribution of organic and inorganic surface species while also hinting at the presence of aragonite at the dorsal protrusion region of the *Eudicella* gralli head, in line with its biological function.

## Abstract (web summary)

Biology serves as inspiration in materials development; this requires improved understanding of the surface chemistry responsible for processes which are being mimicked. Here, the authors report on the use of near edge X-ray absorption fine structure (NEXAFS) imaging to analyze the surface chemistry of insect cuticle.

## Introduction

The arthropod cuticle is a multilayered structure consisting of three main layers: epicuticle (the outermost layer), exocuticle and endocuticle (the innermost layer)^1,2^. The epicuticle acts as a barrier against desiccation and in general acts as a protective layer for the underlying cuticle. The outermost surface of the epicuticle is coated by the cement layer, which is comprised of waxes, lipids and shellac-like cement. However, details of the cement layer, its architecture, chemical composition and spatial organization are unknown. Since the cement layer is in direct contact with the environment, it is extremely important for arthropod survival. Many surface-related effects, such as wettability, water permeability, dirt repellence, frictional resistance and antibacterial properties all depend on the chemistry and ultra-structure of the outermost layer. Understanding the epicuticle surface structure and properties is, therefore, important for both our understanding of arthropod biology and for biomimetic designs. Biomimetic routes might be fruitful for new strategies in material sciences for the development of new anti-adhesive systems, wear-resistant materials, or surfaces with mechano-sensing abilities and specific optical properties.

Studies of the insect cuticle typically follow two orthogonal approaches. The biochemical approach describes the chemistry of the cuticle. This approach typically provides little information about spatial organization of this material and is not surface-specific. Organismal biology, on the other hand, uses microscopy imaging techniques, such as fluorescence light microscopy^3^, confocal laser scanning microscopy^4^ and electron microscopy^5,6^ in combination with mechanical and physical studies^7–11^, to capture information about the cuticle structure, its chemical composition at the microscale, and its properties in detail. However, since these methods are missing the chemical sensitivity and sub-nanoscale resolution, the use of other methods will be required.

**Fig. 1:**
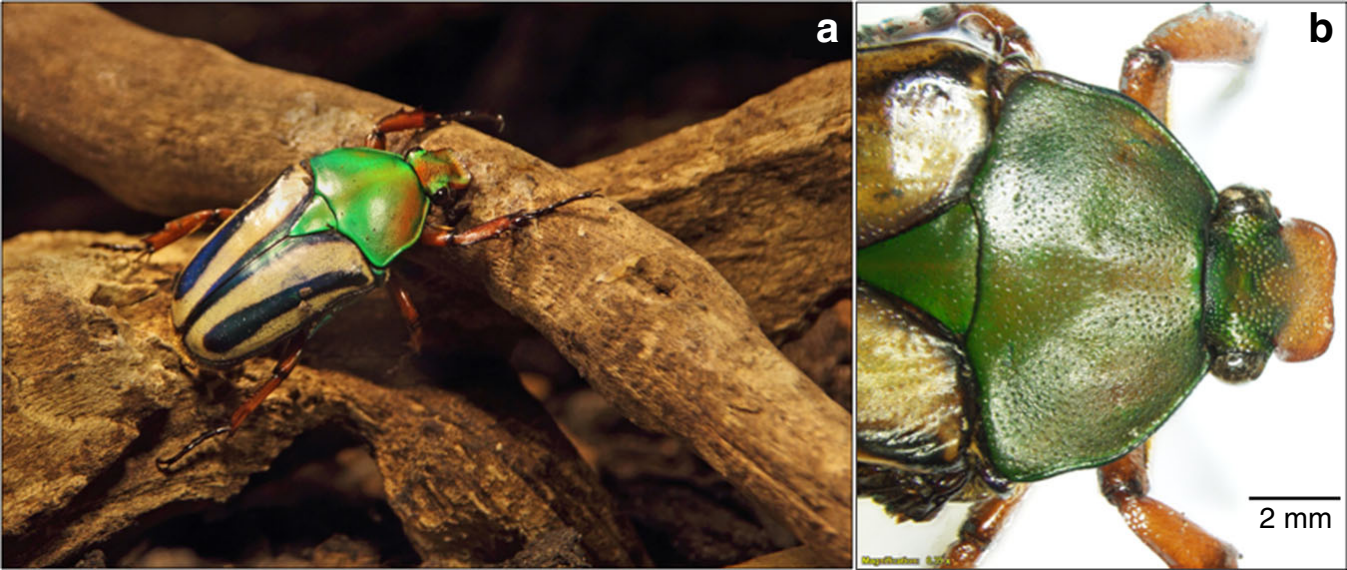
The African Flower Scarab (*Eudicella gralli*). **a** Photograph of a female Flower Scarab. The iridescent coloration is caused by optically active layers within the cuticle. Spectroscopic investigation of the surface chemistry of the scarab cuticle is challenging, because of the curvature and multilayered complexity of the material. **b** Details of the front part of *E. gralli* and the dorsal head protrusion used to dig through soil

While *Morpho* butterflies have long been the focus of the biomimetics of photonic structures, equally interesting and important optical structures have been observed in a diverse variety of beetle species, for example with complex photonic crystals analogous to the fiber-optic technology used to deliver fast data transfer^12,13^. It has been shown that the iridescence in the African flower beetle *Eudicella gralli* (*E. gralli*, Scarabaeidae, Cetonini) cuticle, shown in Fig. 1, is caused by the layered structures of chitin fibers and protein matrix with different refractive indices and angle-dependent reflection of different wavelengths of light. However, the photonic structures can be covered and protected by the epicuticular cement and wax layers^14,15^, which represent a crucial component of the optically active material, and the information about epicuticular coverage of the beetle exoskeleton remains largely unknown.

As laid out above, effective epicuticle imaging techniques must combine chemical specificity with surface sensitivity. Electron spectro-microscopic methods, such as X-ray photoemission electron microscopy (XPEEM)^16^, scanning photoemission microscopy (SPEM) and scanning transmission X-ray microscopy (STXM) hold great promise in this regard. They combine imaging capabilities with sub-micrometer to nanometer lateral resolution and high surface sensitivity with the chemical sensitivity of near edge X-ray absorption fine structure (NEXAFS) spectra^17^. By probing the resonant photoexcitation of atomic core level electrons into unoccupied molecular orbitals, NEXAFS spectroscopy can detect the chemistry of species at the outermost 1–10 nm of material and provide detailed information about the elemental composition, chemical bonds and molecular orientation.

NEXAFS has been developed to provide a detailed analysis of biological surfaces and has been used to track the binding, orientation and structure of proteins^18,19^ and DNA^20^ when attached to surfaces, but also biominerals^21^, hard and soft biological tissue^22,23^, as well as the surface chemistry of snake scales^24^ and frog tongue mucus^25^. STXM and XPEEM both can record NEXAFS spectra with high spatial resolution up to several tens of nanometers and have been used to study protein assembly at biomaterial surfaces and the mineralization of biological tissue^26,27^.

For the analysis of insect epicuticle, the main limitation of STXM is that it is in principle a bulk sensitive transmission method. STXM has been used to probe biomolecules on surfaces. However, in these experiments, the surface sensitivity comes from the extremely thin, X-ray transparent substrates used and from the fact that adsorbate molecules and substrate chemistry are designed to allow their spectroscopic identification. For studies of real insect cuticle, it would be challenging to produce thin, homogeneous samples and the identification of bulk and surface species would be more difficult compared with artificial model system. Unlike STXM, XPEEM does not require X-ray transparent samples. However, XPEEM’s narrow focal depth makes it sensitive to sample curvature and roughness and therefore not ideally suited to interrogate strongly structured samples. Both XPEEM and STXM have high spatial resolution, but are limited to sample surface areas of a few square millimeters.

SPEM can, in principle, analyze large sample areas and can provide surface photoelectron spectra and images of the intensity distribution of surface species^28^. For SPEM, the X-rays are focused onto the surface and the emitted photoelectrons are directed into an energy-dispersive hemispherical analyzer. The spatial information is provided by scanning the sample across the region of interest. The shallow field of depth and the electron lensing make it difficult to image rough and 3D structures. Additionally, insulating biological samples can cause significant spectral distortions due to charging effects, while the high X-ray doses, required for SPEM, can cause severe radiation damage in soft-matter samples.

**Fig. 2:**
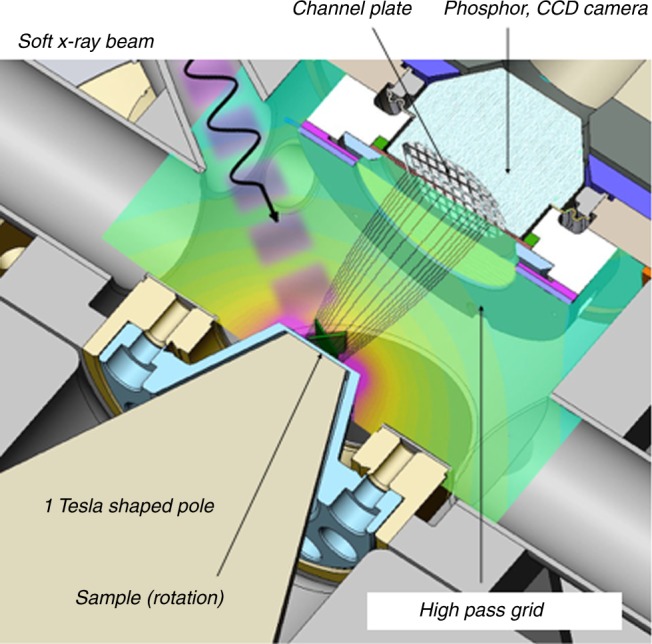
Schematic of the NEXAFS microscope. The system uses magnetic projection electron imaging with a nearly 100% collection efficiency and a large depth of field, which allows the analysis of corrugated samples and sample rotation. Auger electrons are forced to follow the magnetic field lines on helical orbits and analyzed by a grid energy analyzer and detected by phosphor screen and a CCD camera

The challenges posed by imaging insect cuticle are met in by a recently developed NEXAFS microscope (Synchrotron Radiation Instrumentation Inc.) based on magnetic projection electron imaging (see Fig. 2). Guiding photoelectrons to the detector using a magnetic field has several advantages over more conventional electric lensing for the observation of complex, rough and insulating animal tissue. The NEXAFS microscope has no lenses in the electron optical path and the electrons are confined to the magnetic field lines, which are designed to be homogenous over macroscopic sample areas, and are directly imaged onto a large area channel-plate with nearly no distortion over several centimeters. The large depth field allows curved samples to be easily imaged. The lateral resolution of 5 μm is significantly lower than that of XPEEM or STXM. The large field of view, however, allows an overview of large areas of insect surfaces and the analysis of large samples up to 2 cm^2^ in a single image^29,30^.

Here, we report a case study of the epicuticular surface of the African flower beetle *E. gralli*, a brightly colored scarab, with an iridescent rainbow-colored exoskeleton that scatters ambient light and gives a greenish multicolored tint (Fig. 1). The analysis shows the distribution of organic and inorganic surface species. Interestingly, the data hint at the presence of aragonite at the dorsal protrusion region of the *E.* *gralli* head, in line with its biological function as a ‘shovel-like’ device, which the beetle uses to dig through plant tissue and soil.

## Results

### NEXAFS images of the surface of the African flower beetle

**Fig. 3:**
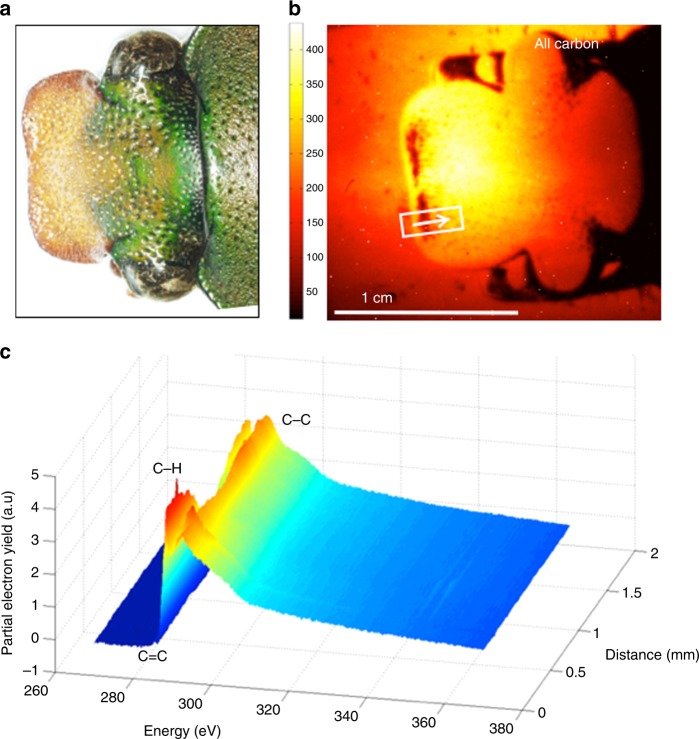
Imaging of an *E. gralli* head at the carbon K-edge. **a** Photograph of the head of flower scarab. **b** NEXAFS image of scarab head. The image is representative of the electron yield across the carbon region 270–370 eV. Each pixel contains a full NEXAFS spectrum. **c** NEXAFS spectra extracted from the image along the line indicated in the image

A NEXAFS microscope was used to probe the surface chemistry of the highly structured, curved and inhomogeneous cuticle of the African flower scarab. The microscopic view of the head of a female *E. gralli* in Fig. 3a shows that the shovel-like protrusion of the head, eyes as well as little dot-like depressions spread over the head harboring mechano-sensory devices. A coloration gradient is going from the thorax toward the mouth, likely caused by different architectures of the cuticle. Panel b shows a NEXAFS carbon K-edge image of an entire scarab head. The image was recorded using a retardation voltage of 50 V, leading to a probing depth of ~5 nm^31^. The image is representative of the Auger electron spectrum across the entire carbon region from 270 to 370 eV after pre- and post-edge normalization to an edge jump of unity. The NEXAFS signal is rather homogeneous across the head with somewhat lower signal near the thorax and for the two indentations near the dorsal head protrusion. The lower signals are likely explained by shadowing effects. Interestingly, the curvature of the head does not significantly affect the signal intensity. Each pixel within the image contains a full NEXAFS spectrum and spectra can be extracted from the images to highlight variations in surface chemistry. Figure 3c shows carbon spectra across the white line indicated in the NEXAFS image. The spectra contain a weak pre-edge feature near 285 eV related to aromatic species as well as resonances related to C–H, C=O and C–C bonds near 286 eV, 288 eV and above 290 eV.

**Fig. 4:**
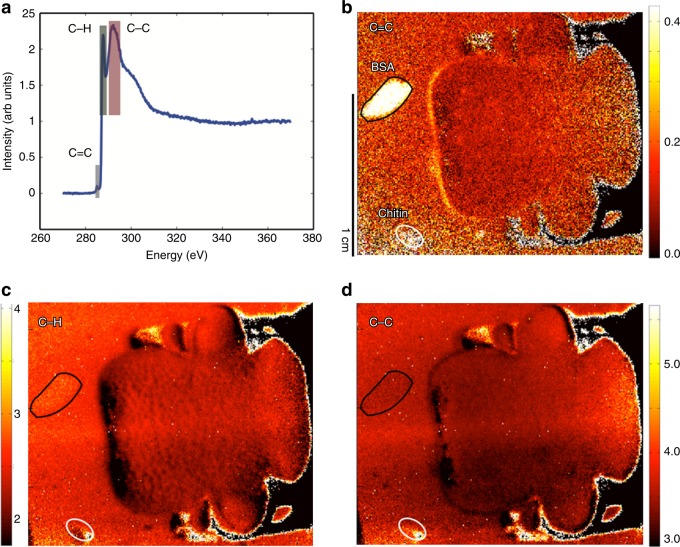
NEXAFS images of the scarab head at different resonance energies. **a** NEXAFS carbon spectra averaged across the entire sample. Energy regions for the extracted images related to specific regions are indicated. **b** NEXAFS image related to photoemission near 285 eV. This spectral range corresponds to aromatic C = C bonds. **c** Image related to the C–H region near 286–290 eV. **d** Image representative for C–C bonds in the region from 290 to 297 eV. While the C–C bonds are evenly distributed across the scarab head, C–H and aromatic species shows spatial variation. All spectra and images are pre- and post-edge normalized and therefore representative of the number of the respective bonds with respect to the total amount of carbon. Areas marked in black or white indicate the position of albumin (top) and chitin (bottom) reference materials. All images and spectra are pre- and post-edge normalized

This analysis provides information about variations in surface chemistry across the very surface of the biological sample. Images based on specific regions of the spectrum can highlight the distribution of spectral features, i.e. chemical bonds, across the tissue sample surface. Figure 4 displays images based on three different characteristic regions near the carbon K-edge as highlighted in panel a. It is important to note that the spectral data are normalized to the pre-and post-edge and representative of the number of carbon bonds relative to the total amount of carbon^17^. Panel b shows an image representative of aromatic carbon double bonds, such as aromatic rings and in-chain species near 285 eV^32^. In the biological material, these are, e.g., found in proteins and unsaturated lipids^19^. The signal observed across the beetle head is relatively weak and evenly distributed except for strong signal near the rim of the dorsal protrusion. This indicates a variation of the surface chemistry near the edge of the head.

For an internal reference, the sample also contained a spotted bovine serum albumin (BSA) and a chitin (see areas marked in Fig. 4 and extracted spectra in the supporting information document, Supplementary Figs. 1 and 2). The NEXAFS images show a strong signal related to the protein sample (region 1) but the chitin emission was similar to the sample holder background, which is visible next to the beetle head. The similarity to the spotted protein sample indicates the strong signal is likely related to aromatic protein side chains, very similar to the edge region of the scarab head^16,33^.

Panel c displays variations across the sample related to C–H bonds, which are abundant in waxes and other lipids^17^. Here, the punctae display a somewhat reduced C–H content. Since the C–C and C=C signal does not vary across the head area, the signal variation across the punctae cannot be explained by curvature or edge effects. Obviously, the cement layer surface within the punctae is markedly different from the surrounding epicuticular surface, likely with lower hydrocarbon content.

**Fig. 5:**
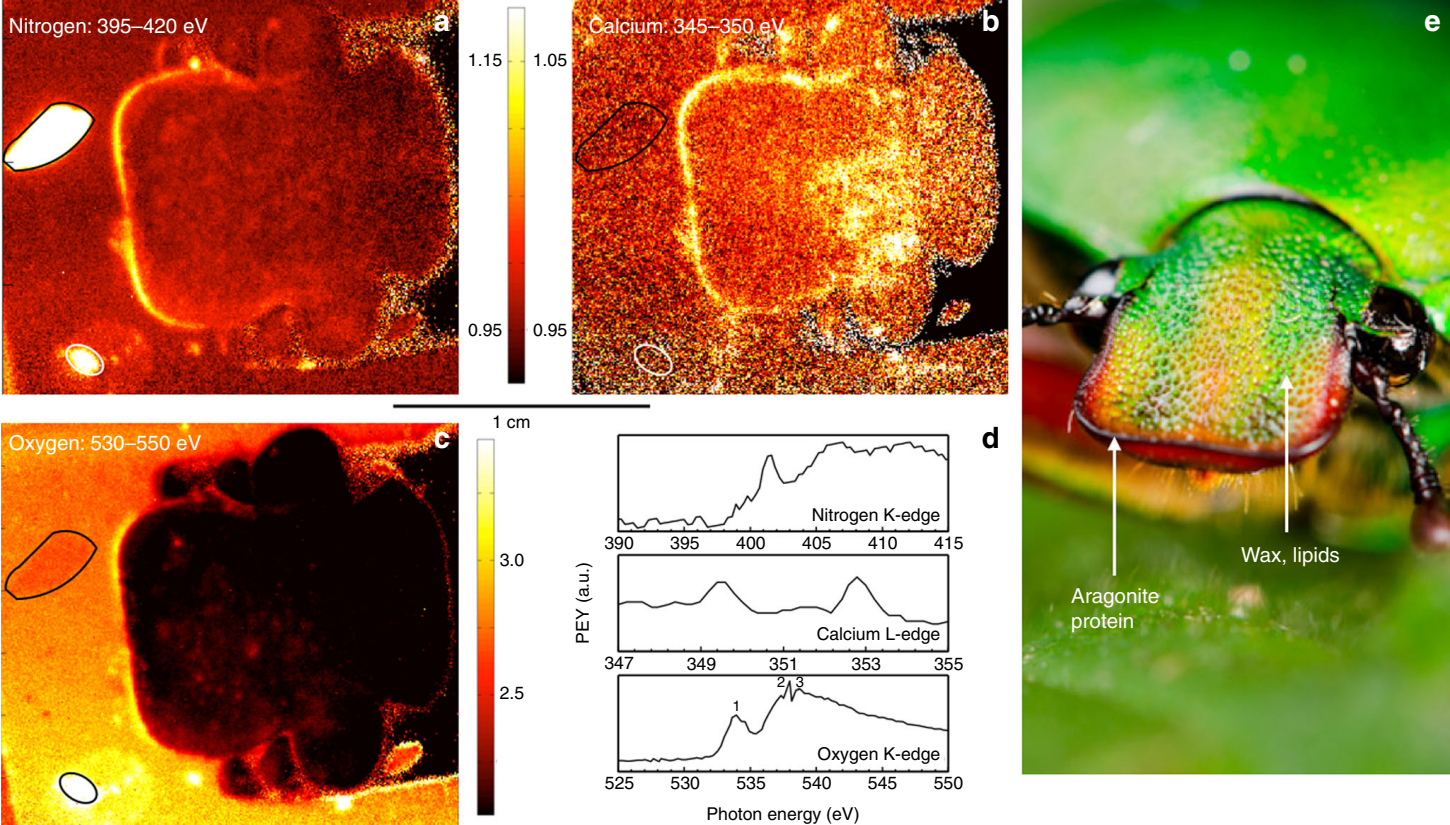
Spectral analysis of the dorsal protrusion region. **a** NEXAFS image related to the nitrogen K-edge region at 395–420 eV. This spectral range corresponds to nitrogen bonds at the surface. **b** Image related to the calcium L-edge (345–350 eV). The presence of both nitrogen and calcium may be interpreted as biomineralized tissue near the scarab dorsal protrusion of the head. **c** Oxygen NEXAFS image in the 530–550 eV region. **d** Spectra extracted from the dorsal protrusion of the head. Areas marked in black or white indicate the position of albumin (top) and chitin (bottom) reference materials. All images and spectra are pre-edge normalized. **e** Photograph highlighting the surface chemistries found on the head of *E. gralli*

To shed light on the question what is the origin of the signal variations in the carbon region, we also recorded nitrogen K-edge images, which can differentiate between fatty acids, chitin and proteinaceous components^16,33^. The nitrogen NEXAFS images shown in Fig. 5a clearly demonstrate strong signal near the dorsal head protrusion. As expected, the reference protein and chitin spots also show significant nitrogen signal. The spectra extracted from the protrusion region (see rectangular region of interest Fig. 3) show intense amide π* resonances, a signature typical for proteins and chitin (Fig. 5d)^18,19^.

Surprisingly, the same region shows appreciable amounts of calcium, as can be seen in the calcium L-edge image in Fig. 5b and the spectra extracted from the protrusion region (Fig. 5d). Here the rim of the mouthpart (Fig. 5e) shows a clear line of calcium-rich tissue. In combination with the strong nitrogen signal and the aromatic carbon bonds also found near the rim, this indicates that the dorsal protrusion region may be lined with a biomineralized edge of calcium-containing cuticle.

**Fig. 6:**
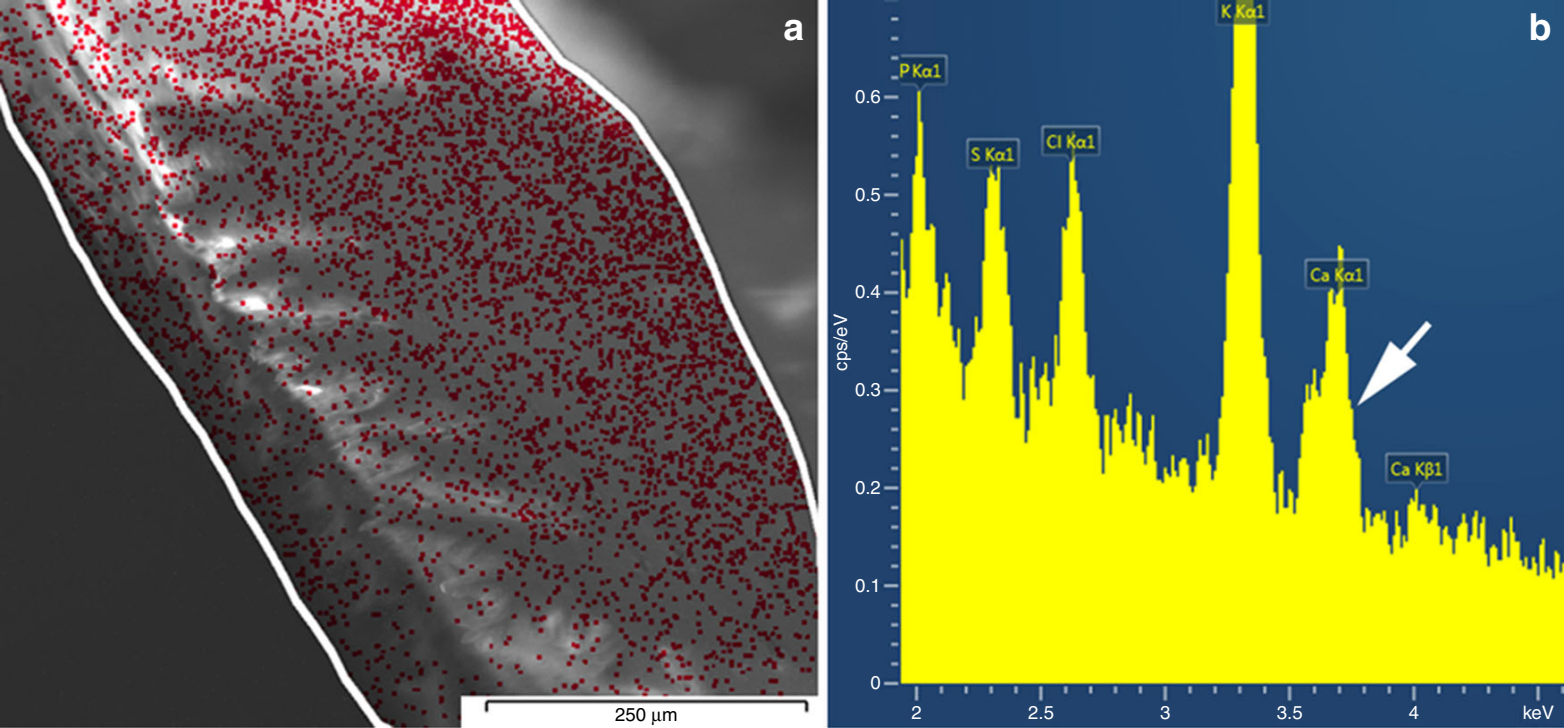
SEM image and spatial distribution of Ca along lateral head margin. **a** EDX analysis of the spatial distribution of Ca. **b** Typical EDX spectrum showing the presence of Ca (0.02–0.04 atomic %)

Insect cuticle is also exposed to severe mechanical strain and abrasion. *E. gralli* uses the dorsal protrusion of the head to dig through soil and wood and the question arises how the cuticle is strengthened in these areas. While extended reinforcement by biominerals have only rarely been observed in beetle tissue^34^, microscopic areas of mineralized tissue, which can reinforce the epicuticle against mechanical strain would be extremely difficult to detect with most conventional techniques. Therefore, to estimate the extend of biomineralization and the thickness of the layer, we performed an elemental analysis using energy-dispersive X-ray (EDX) spectroscopy. EDX is a standard in the chemical analysis of various biological surfaces. Additionally, the combination of EDX and NEXAFS can provide some qualitative information about concentration and the depth at which the elements are deposited in the specimen: NEXAFS probes the outermost surface while EDX is a bulk sensitive method. Figure 6 (right panel) shows a scanning electron microscopy (SEM) image of the dorsal protrusion along with the location where EDX spectra were taken. The spectra, shown in Fig. 6 (left panel), show all the elements expected for insect cuticle, including some metals. Calcium is detected only in trace amounts near 0.04 ± 0.02 (standard deviation) atom percent. This leaves two scenarios: calcium could be dispersed throughout the entire probing depth of EDX of around 1 μm or it could be concentrated at the cuticular surface. Since the NEXAFS signal observed for calcium is very strong, the latter scenario is more likely here. When renormalizing the calcium content to a thin interfacial biomineral layer with a 10% calcium concentration, the thickness of that layer can be estimated to be ~10 nm.

To identify the type of biomineral, we considered three calcium-based minerals typically found in biology: calcium carbonate, -phosphate and -oxalate. Rajendran et al. have published reference calcium NEXAFS spectra for different minerals^35^. The study shows that calcium L-edge spectra for these biominerals are very similar and not ideal to identify the type of mineral^35^. Oxygen K-edge spectra are more sensitive to the type of calcium-based mineral^36^. The pre-edge normalized oxygen image in Fig. 5c displays the distribution of oxygen on the beetle head. A spectrum extracted from the protrusion region (panel d) exhibits a significant pre-edge feature related to a C=O π* resonance (peak 1) near 539.9 eV along with σ* resonances near 537.2 eV and 538.7 eV (peaks 2 and 3, respectively).

When comparing with published spectra for calcium phosphates, such as hydroxyapatite and tricalcium phosphate, these materials can be ruled out since the spectra lack a 534eV pre-edge feature related to a C=O bond^35^. Calcium oxalates and -carbonates both show pronounced pre-edge C=O π* resonances^35,37^. Comparison with published spectra for metal coordinated oxalic acid shows that, while oxalate also has a strong pre-edge C=O π*, the energy positions of the σ* resonances (peaks 2 and 3) are visible at 543 eV and 546 eV—at significantly higher photon energies compared with the spectra observed in our study^37^.

Calcium carbonates generally match the observed spectra better. As shown in a detailed study of calcium carbonates by DeVol et al., the energy positions of the σ* resonances vary significantly for the different phases of calcium carbonate^36^. While the energy position of peak 2 matches for all phases except the hydrated amorphous phase, a feature near 538.7 eV is observed for aragonite, but is missing for vaterite, calcite, and amorphous phases of calcium carbonate^36^. A higher energy resonance near 545 eV observed by DeVol et al. is strongly angle dependent and less pronounced, but visible in the cuticle spectra^36^. Ostensibly, the protrusion area near the edge of the head is lined with a nanometer-thin layer of aragonite.

While calcium carbonate mineralization among beetle is very rare, it has been observed for Coleoptera^34^. Colonial hard coral polyps also deposit calcium carbonate as the aragonite polymorph, stabilized into a continuous calcareous skeleton^38^. Some fossil evidences and molecular biology data suggest that aragonite shell is more ancient than calcite shell for the Bivalvia^39^. Bryozoan skeletons are mineralogically variable, and can be entirely calcitic, entirely aragonitic or bimineralic^40,41^. Ediacaran *Cloudina* and *Namacalathus*, which are among the earliest shell-forming organisms, originally produced aragonitic skeletons, which later underwent diagenetic conversion to calcite^42^. Crystals of aragonite have been also revealed within Codiacean algae^43^, which means that the origin of aragonite biomineralization should be at the level of branching between animals and plants.

Among arthropods, cuticle calcification is found in crustaceans and millipedes, and in a very small number of insects, such as calcified puparium of *Musca autumnalis*^1^. Calcified cuticles provide extra hardness, and in Crustacea, calcification provides mechanical stability enabling them to resist strong hydrostatic pressures underwater. Mineralization is presumably so rarely observed in adult stages of insects, because it would make them too heavy for flying. The minerals involved in the cuticle calcification are calcite, vaterite^44^ and hydroxyapatite^45^. The mineral content may be rather high (up to 55% in some millipedes)^46^. In crustacean, calcification occurs in the inner epicuticle, the exocuticle and the endocuticle, but not in the so-called helicoidal membranous layer between the rest of the endocuticle and the epidermis^1^. In our study, we clearly demonstrate the presence of calcium in the beetle epicuticle, but not in the deeper layers of cuticle. This very targeted way of calcification may provide an additional hardness and wear resistance to the cuticle without adding much extra weight to the exoskeleton of the flying insect. Our finding of aragonite in insects further supports rather general occurrence of this calcium carbonate form among living organisms.

The surface-related effects of the calcified layer remain unclear and certainly deserve separate studies. It is hard to predict how this layer may influence wettability, water permeability, dirt repellence or antibacterial properties. However, we can hypothesize that since hardness/stiffness of the calcified superficial layer is high, it might increase wear resistance (frictional resistance) of the surface. The presence of aragonite hardened tissue in *E. gralli* would be well in line with the biological function of the dorsal protrusion as a ‘shovel-like’ device, which females of *E. gralli* uses to dig through wood.

### NEXAFS microscopy as an analytical tool in organismal biology

NEXAFS microscopy will be helpful in understanding the role of the molecular structure of the outermost surface of insect cuticle. For example, understanding the mechanisms used to control wetting, bacterial adhesion, material hardening and optical patterning could improve our understanding of insect biology and aid attempts in material science to mimic desirable properties of insect tissue. In addition, the comparison with EDX data highlights the sensitivity of NEXAFS imaging for thin interfacial layers that are impossible to track with more bulk sensitive methods. The ability to image the outermost nanometers of macroscopic areas of real-life tissue samples will be a breakthrough in organismal biology in general and open up approaches to bring molecular surface science into the field.

## Methods

### NEXAFS microscopy

NEXAFS spectra were collected at the National Synchrotron Light Source (NSLS) beamline U7A at Brookhaven National Laboratory using a parallel processing imaging system^47^. A soft x-ray beam, with energy scanned around the carbon K-edge (270–340 eV; resolution ~0.1 eV; flux ~5 × 10^10^ photons/s), was rastered across an 18 × 13 mm^2^ area on the sample. This NEXAFS microscope is an instrument known as the Large Area Rapid Imaging Analytical Tool (LARIAT), manufactured by Synchrotron Radiation Instrumentation Inc. The LARIAT was used to measure Auger electrons in Partial Electron Yield (PEY). Step size for the carbon K-edge scans was 0.1 eV with a 2-s dwell time. The time required for the entire scan is <24 min. The emitted photoelectrons were guided to an electron yield detector by a full field imaging parallel magnetic field. The detection scheme provides an almost 100% detection efficiency, a large depth of detectable field and a reduced sample charging by the use of the returning rejected electrons back to the sample. This produced a series of two dimensional NEXAFS images with a 50-μm spatial resolution^47^. To eliminate the effect of incident beam intensity fluctuations and absorption features in the beamline optics, the PEY signals were normalized by the photo yield of a clean gold mesh located upstream along the path of the incident X-ray beam. The experiments have been repeated with a second individual and different acquisition angles. All images were pre- and post-edge normalized to an edge jump of unity. The calcium L-edge images were prepared by plotting the area of the Ca L_2_-edge emission. Beetle samples were provided by the Woodland Park Zoo in Seattle, WA, and all ethical regulations were complied with.

### EDX spectroscopy

The beetle head cuticle was investigated by SEM on a Zeiss Gemini Ultra 55 Plus. Elementary analysis was carried out with the EDX Oxford x-act 10 mm^2^ Silicon Drift Detector accessory of the SEM instrument and AZtecOne software. The dray cuticle samples were uncoated. Following parameters were applied: measurement time 113.7 s, accelerating voltage 20.00 kV, magnification × 300–350, working distance 6.4 mm.

## Supplementary information

Supplementary Information

## Data Availability

All NEXAFS imaging data are available from the authors upon request and on figshare under https://figshare.com/s/26e6744a7b883dacdc3f

## References

[1] Neville, A. C. *Biology of the Arthropod Cuticle* (Springer, Berlin, 1975).

[2] Hepburn, H. R. *Structure of the Integument* (Pergamon Press, 1985).

[3] Gorb SN (1999). Serial elastic elements in the damselfly wing: mobile vein joints contain resilin. Naturwissenschaften.

[4] Michels J, Gorb SN (2012). Detailed three-dimensional visualization of resilin in the exoskeleton of arthropods using confocal laser scanning microscopy. J. Microsc..

[5] Gorb, S. *Attachment Devices of the Insect Cuticle* (Kluwer Academic Publishers, 2001).

[6] Schwarz H, Gorb S (2003). Method of platinum-carbon coating of ultrathin sections for transmission and scanning electron microscopy: an application for study of biological composites. Microsc. Res.Tech..

[7] Arzt E, Enders S, Gorb S (2002). Towards a micromechanical understanding of biological surface devices. Z. Metallkd..

[8] Klocke D, Schmitz H (2011). Water as a major modulator of the mechanical properties of insect cuticle. Acta Biomater..

[9] Peisker H, Michels J, Gorb SN (2013). Evidence for a material gradient in the adhesive tarsal setae of the ladybird beetle Coccinella septempunctata. Nat. Commun..

[10] Wang LY, Rajabi H, Ghoroubi N, Lin CP, Gorb SN (2018). Biomechanical strategies underlying the robust body armour of an aposematic weevil. Front. Physiol..

[11] Eshghi S, Jafarpour M, Darvizeh A, Gorb SN, Rajabi H (2018). A simple, high-resolution, non-destructive method for determining the spatial gradient of the elastic modulus of insect cuticle. J. R. Soc. Interface.

[12] Parker AR (2009). Natural photonics for industrial inspiration. Philos. Trans. R. Soc. A.

[13] Galusha JW, Richey LR, Gardner JS, Cha JN, Bartl MH (2008). Discovery of a diamond-based photonic crystal structure in beetle scales. Phys. Rev. E.

[14] Sun MX (2012). Compound microstructures and wax layer of beetle elytral surfaces and their influence on wetting properties. PLoS ONE.

[15] Lenau T, Barfoed M (2008). Colours and metallic sheen in beetle shells—a biomimetic search for material structuring principles causing light interference. Adv. Eng. Mater..

[16] Leung BO (2012). Using X-PEEM to study biomaterials: protein and peptide adsorption to a polystyrene-poly(methyl methacrylate)-b-polyacrylic acid blend. J. Electron. Spectrosc..

[17] Stöhr, J. *NEXAFS Spectroscopy* (Springer-Verlag, 1992).

[18] Liu XS (2006). Characterization of protein immobilization at silver surfaces by near edge X-ray absorption fine structure spectroscopy. Langmuir.

[19] Weidner T, Apte J, Gamble LJ, Castner DG (2010). Probing the orientation and conformation of α-helix and β-strand model peptides on self-assembled monolayers using sum frequency generation and NEXAFS spectroscopy. Langmuir.

[20] Lee CY, Nguyen PCT, Grainger DW, Gamble LJ, Castner DG (2007). Structure and DNA hybridization properties of mixed nucleic acid/maleimide-ethylene glycol monolayers. Anal. Chem..

[21] Metzler, R. A. et al. XANES in nanobiology. in *X-Ray Absorption Fine Structure-Xafs13* (eds) (American Institute of Physics, 2007).

[22] Weidner T (2012). Direct observation of phenylalanine orientations in statherin bound to hydroxyapatite surfaces. J. Am. Chem. Soc..

[23] Baio JE (2010). Multitechnique characterization of adsorbed peptide and protein orientation: LK3(10) and Protein G B1. J. Vac. Sci. Technol. B.

[24] Baio JE (2015). Evidence of a molecular boundary lubricant at snakeskin surfaces. J. R. Soc. Interface.

[25] Fowler JE (2018). Surface chemistry of the frog sticky-tongue mechanism. Biointerphases.

[26] Ma Y (2009). The grinding tip of the sea urchin tooth: exquisite control over calcite crsytal orientation and Mg distribution. Proc. Natl Acad. Sci. USA.

[27] Gong YUT (2012). Phase transitions in biogenic amorphous calcium carbonate. Proc. Natl Acad. Sci. USA.

[28] Kazemian Abyaneh M, Gregoratti L, Amati M, Dalmiglio M, Kiskinova M (2011). Scanning photoelectron microscopy: a powerful technique for probing micro and nano-structures. e-J. Surf. Sci. Nanotechnol..

[29] Baio JE, Jaye C, Fischer DA, Weidner T (2013). Multiplexed orientation and structure analysis by imaging near-edge X-ray absorption fine structure (MOSAIX) for combinatorial surface science. Anal. Chem..

[30] Baio JE, Jaye C, Fischer DA, Weidner T (2014). High-throughput analysis of molecular orientation on surfaces by NEXAFS imaging of curved sample arrays. ACS Comb. Sci..

[31] Sohn KE (2009). Determination of the electron escape depth for NEXAFS spectroscopy. Langmuir.

[32] Frey S (2001). Structure of thioaromatic self-assembled monolayers on gold and silver. Langmuir.

[33] Zubavichus Y, Shaporenko A, Grunze M, Zharnikov M (2008). Is X-ray absorption spectroscopy sensitive to the amino acid composition of functional proteins?. J. Phys. Chem. B.

[34] Leschen RAB, Cutler B (1994). Cuticular calcium in beetles (Coleoptera, Tenebrionidae, Phrenapetinae). Ann. Entomol. Soc. Am..

[35] Rajendran J, Gialanella S, Aswath PB (2013). XANES analysis of dried and calcined bones. Mater. Sci. Eng. C.

[36] DeVol RT (2014). Oxygen spectroscopy and polarization-dependent imaging contrast (PIC)-mapping of calcium carbonate minerals and biominerals. J. Phys. Chem. B.

[37] White TW (2018). A structural investigation of the interaction of oxalic acid with Cu(110). Surf. Sci..

[38] Domart-Coulon IJ, Elbert DC, Scully EP, Calimlim PS, Ostrander GK (2001). Aragonite crystallization in primary cell cultures of multicellular isolates from a hard coral, Pocillopora damicornis. Proc. Natl Acad. Sci. USA.

[39] Wang XT (2014). Aragonite shells are more ancient than calcite ones in bivalves: new evidence based on omics. Mol. Biol. Rep..

[40] Taylor PD, Kudryavtsev AB, Schopf JW (2008). Calcite and aragonite distributions in the skeletons of bimineralic bryozoans as revealed by Raman spectroscopy. Invertebr. Biol..

[41] Loxton J, Jones MS, Najorka J, Smith AM, Porter JS (2018). Skeletal carbonate mineralogy of Scottish bryozoans. PLoS ONE.

[42] Pruss SB, Blattler CL, Macdonald FA, Higgins JA (2018). Calcium isotope evidence that the earliest metazoan biomineralizers formed aragonite shells. Geology.

[43] Perkins RD, Mckenzie MD, Blackwelder PL (1972). Aragonite crystals within Codiacean algae—distinctive morphology and sedimentary implications. Science.

[44] Dudich E (1931). Systematische und biologische Untersuchungen über die Kalkeinlagerungen des Crustaceenpanzers in polarisiertem Licht. Zoologica.

[45] Needham AE (1954). Properties of the minerals in the exuvia of Crustacea. Quart. J. Microsc. Sci..

[46] Shrivastava SC (1970). Cuticular components of common Indian arachnids and myriapods. Experientia.

[47] Konicek AR (2011). Near-edge X-ray absorption fine structure imaging of spherical and flat counterfaces of ultrananocrystalline diamond tribological contacts: a correlation of surface chemistry and friction. Tribol. Lett..

