# Supplementary material for: NEXAFS imaging to characterize the physio-chemical composition of cuticle from African Flower Scarab Eudicella gralli
Source: Nat Commun. 2019 Oct 18;10:4758. doi: 10.1038/s41467-019-12616-5 (PMC6802387; doi:10.1038/s41467-019-12616-5)
Supplement: Supplementary file 1 — Supplementary Information [file 41467_2019_12616_MOESM1_ESM.pdf]

Supplementary Information:

NEXAFS imaging to characterize the physio-chemical compostion of cuticle from African Flower Scarab *Eudicella gralli*

Joe E. Baio, Cherno Jaye, Erin Sullivan, Mette H. Rasmussen, Daniel A. Fischer, Stanislav Gorb, Tobias Weidner

Supplementary Figures.

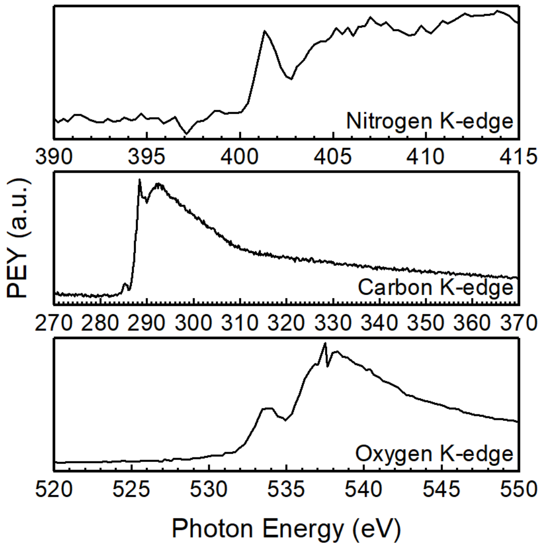

Supplementary Figure 1: NEXAFS spectra extracted from the images for the chitin sample spotted into the sample holder as reference material.

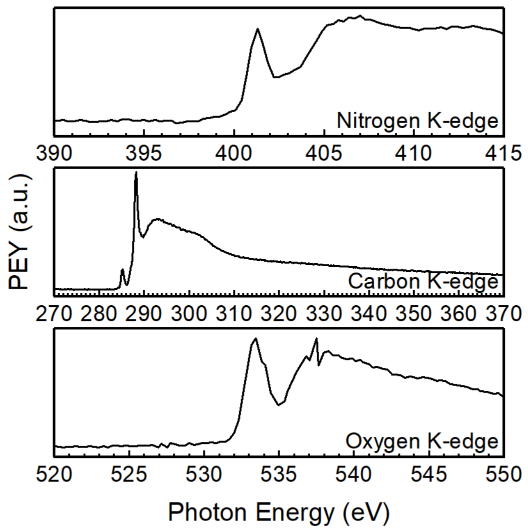

Supplementary Figure 2: NEXAFS spectra extracted from the images for the bovine serum albumin sample spotted into the sample holder as reference material.
